# Tumor Microenvironment Lactate: Is It a Cancer Progression Marker, Immunosuppressant, and Therapeutic Target?

**DOI:** 10.3390/molecules30081763

**Published:** 2025-04-15

**Authors:** Eugene Y. Kim, Joyce Abides, Chandler R. Keller, Steve R. Martinez, Weimin Li

**Affiliations:** 1 Department of Translational Medicine and Physiology, Elson S. Floyd College of Medicine, Washington State University, Spokane, WA 99202, USA; eugene.y.kim@wsu.edu (E.Y.K.); joyce.abides@wsu.edu (J.A.); chandler.keller@wsu.edu (C.R.K.); 2 Doctor of Medicine Program, Elson S. Floyd College of Medicine, Washington State University, Spokane, WA 99202, USA; 3 Department of Medical Education and Clinical Sciences, Elson S. Floyd College of Medicine, Washington State University, Spokane, WA 99202, USA; 4 Providence Regional Cancer Partnership, Providence Regional Medical Center, Everett, WA 98201, USA

**Keywords:** lactate, tumor microenvironment (TME), cell metabolism, signaling and gene regulation, posttranslational modification, immune regulation

## Abstract

The “Warburg effect” is a term coined a century ago for the preferential use of glycolysis over aerobic respiration in tumor cells for energy production, even under aerobic conditions. Although this is a less efficient mechanism of generating energy from glucose, aerobic glycolysis, in addition to the canonical anaerobic glycolysis, is an effective means of lactate production. The abundant waste product, lactate, yielded by the dual glycolysis in a tumor, has been discovered to be a major biomolecule that drives cancer progression. Lactate is a metabolic energy source that, via cell membrane lactate transporters, shuttles in and out of cancer cells as well as cancer cell-associated stromal cells and immune cells within the tumor microenvironment (TME). Additionally, lactate serves as a pH tuner, signaling ligand and transducer, epigenetic and gene transcription regulator, TME modifier, immune suppressor, chemoresistance modulator, and prognostic marker. With such broad functionalities, the production–consumption–reproduction of TME lactate fuels tumor growth and dissemination. Here, we elaborate on the lactate sources that contribute to the pool of lactate in the TME, the functions of TME lactate, the influence of the TME lactate on immune cell function and local tissue immunity, and anticancer therapeutic approaches adopting lactate manipulations and their efficacies. By scrutinizing these properties of the TME lactate and others that have been well addressed in the field, it is expected that a better weighing of the influence of the TME lactate on cancer development, progression, prognosis, and therapeutic efficacy can be achieved.

## 1. TME Lactate Sources

### 1.1. The Warburg Effect

Lactate has traditionally been regarded as a waste product of glycolysis, the central metabolic pathway that converts glucose into pyruvate [1]. Under anaerobic conditions, pyruvate is converted to lactate by the enzyme lactate dehydrogenase A (LDHA), which regenerates NAD+, essential for sustaining ATP production during glycolysis [2]. This process is particularly prominent in tissues with high-energy demands and low-oxygen conditions, such as muscle cells during exercise. In normal oxygenated tissue cells, pyruvate is transported to mitochondria, where it generates significantly more ATP through aerobic respiration than via glycolysis. Despite total energy production inefficiency, glycolysis is adopted by cancer cells for ATP generation, even under aerobic conditions. This aerobic glycolysis is known as the “Warburg effect”, named after Otto Warburg, who discovered that glucose uptake significantly increased in cancer cells, leading to the production of excessive amounts of lactate [3]. In a tumor respiration experiment, Warburg and colleagues observed that tumors utilized 66% of circulating glucose to produce lactate [4]. This aberrant glycolytic behavior is part of the tumor survival machinery and is considered a hallmark of cancer [5].

Similar to Warburg’s discovery, using 13C-labeled glucose supplied at human circulating blood levels (1 g/L), breast cancer cells cultured in a native tissue-mimicking 3D TME secreted lactate that had 45% carbon from the isotope-labeled glucose and about 50% carbon from natural 12C-carbon sources [6]. In contrast, lactate secreted from normal breast epithelial cells grown in the same TME only contained 28% carbon sourced from the 13C-labeled glucose. This observation suggests that cancer cells indeed heavily consume TME glucose and convert about half of its imported glucose into lactate for their survival and growth. Furthermore, it is consequential that proliferating normal cells also produce lactate.

### 1.2. The Reverse Warburg Effect

**Figure 1:**
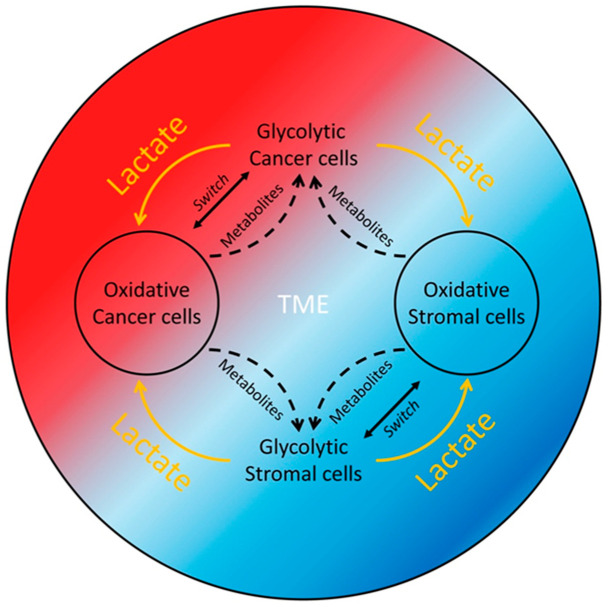
Warburg effect and reverse Warburg effect form a core symbiotic mechanism supporting cancer cell thriving in the TME. Glycolytic cancer cells or stromal cells living in the TME of a growing tumor produce and release lactate for the growth of oxidative cancer cells or stromal cells. In turn, the oxidative cancer cells or stromal cells utilize the imported lactate to generate pyruvate or other metabolites and release them for the survival and propagation of the glycolytic cancer cells or stromal cells.

The metabolic crosstalk between cancer cells and cancer-associated fibroblasts (CAFs) is another critical aspect of TME modification (Figure 1). While the Warburg effect describes cancer cells relying on aerobic glycolysis for energy, the “Reverse Warburg Effect” (lactate shuttle) describes how cancer cells induce a metabolic reprogramming of CAFs through oxidative stress and inflammatory signaling. As a result, CAFs undergo aerobic glycolysis, resulting in lactate production, which subsequently fuels oxidative phosphorylation (OXPHOS) in adjacent cancer cells [7] (Figure 1). This reciprocal lactate shuttle interplay between cancer cells and stromal CAFs was nicely demonstrated by Fiaschi et al. in prostate cancer (PCa) cell and fibroblast culture models, where contact of stromal fibroblasts with PCa cells and oxidative stress and hypoxia-inducible factor 1 subunit alpha (HIF1-α) stabilization within the fibroblasts were required for the Warburg effect’s production of lactate [8]. Furthermore, the study by Curry et al. identified monocarboxylate transporters (MCT1 and MCT4) as key biomarkers driving this metabolic symbiosis [9]. The authors demonstrated that MCT4 was primarily expressed in CAFs, where it aided in the export of lactate generated from aerobic glycolysis. In contrast, MCT1 was found in oxidative tumor cells, facilitating the uptake of lactate, which was then utilized as an energy source through oxidative phosphorylation. This metabolic coupling fosters tumor aggressiveness and enables tumors to adapt to hypoxic and nutrient-deprived conditions.

**Figure 2:**
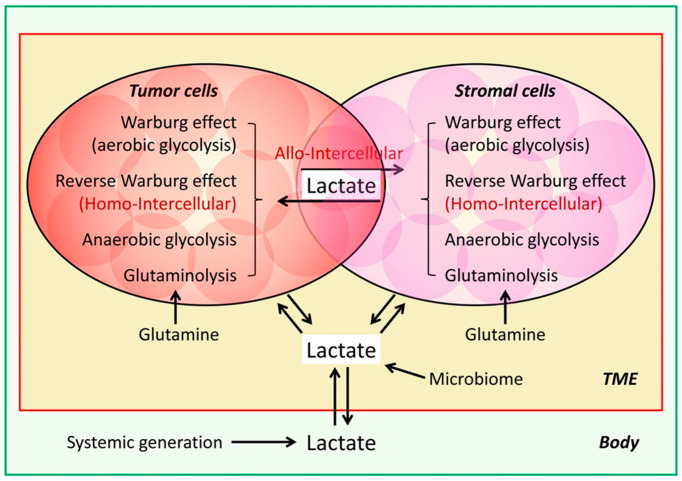
Model of lactate sources supplying the TME for tumor progression. Both tumor cells and stromal cells utilize the Warburg effect, reverse Warburg effect, canonical glycolysis, and glutaminolysis to lactate production. The reverse Warburg effect that happens in the same type of cells that are under either glycolytic or oxidative status is considered a homo-intercellular mechanism, and the Warburg effect that happens between two different types of cells is considered an allo-intercellular mechanism. Tumor-generated lactate and systemically generated circulating lactate are mutually exchangeable and available for tumor growth.

We recently showed that the lactate-depleting bacterial lactate oxidase (LOX) killed proliferating cells, especially cancer cells that grow faster, but not non-proliferating or senescent normal cells [6,10]. These data suggest that any proliferating cells within the TME, including the CAFs described above, could potentially contribute lactate to the TME lactate pool to support cancer cell survival and growth and tumor progression. It is also possible that different cancer cell or stromal cell subpopulations within a tumor may themselves form a lactate production and consumption symbiotic relationship or that they are both lactate producers and consumers (Figure 1). The lactate production and consumption of the interdependent cells of the same type (homo-intercellular reverse Warburg effect) or a different type (allo-intercellular reverse Warburg effect) within a tumor form a perpetual motion mechanism to support the survival and propagation of the cell populations, and, thus, tumor progression (Figure 1 and Figure 2).

Although the process of glycolysis is less efficient in producing ATP than oxidative phosphorylation, glycolysis provides multiple advantages for rapidly dividing tumor cells. For instance, aerobic glycolysis generates ATP much faster compared to OXPHOS, allowing it to meet the high energy demands of proliferating tumor cells [11]. The intermediates of glycolysis can be used by tumor cells to synthesize macromolecules, such as proteins, nucleic acids, and lipids, which are crucial for cell growth and division [12]. Furthermore, the process of anaerobic glycolysis reduces free radical production and consequently decreases toxicity to tumor cells [13]. Importantly and not surprisingly, cancer cells can switch between glycolytic and oxidative states under lactate-induced acidotic conditions [14], resulting in an interlocking mechanism that can effectively cope with stresses arising in the TME (Figure 1).

### 1.3. Glutaminolysis or Activation of Lactate-Generating Enzymes

In addition to the Warburg and reverse Warburg effects, an important source of lactate in cancer cells is glutaminolysis [15]. Glutamine, the most abundant amino acid in the body, enters tumor cells through the amino acid transporter sodium-dependent solute carrier family 1 member 5 (SLC1A5) to serve as an essential cellular nutrient [16]. In mitochondria, glutamine is metabolized by glutaminase (GLS) into glutamate, which can then be converted into α-ketoglutarate that enters the TCA cycle to generate products essential for either maintaining the cycle or producing lactate via the malate to pyruvate route. The increased secretion of lactate by tumors can partially arise from overexpression of insulin-like growth factor 2 mRNA-binding protein 3 (IGF2BP3) as well as loss of serine/threonine kinase 11/liver kinase B1 (STK11/LKB1), possibly in a MCT4-dependent manner [17,18,19]. IGF2BP3 binds to m6A sites on LDHA [17], GLS, and glutamate dehydrogenase 1 (GLUD1) mRNAs [18], increasing their longevity and either directly increasing lactate production or facilitating glutamate and glutamine metabolism to positively reinforce its production.

### 1.4. Tumor Microbiome

Various microbiotas have been identified in human tumors of different organs, such as the colon, stomach, liver, pancreas, lung, cervix, and ovary, which can produce lactate and directly or indirectly promote cancer progression. For example, resident microbiota *Lactobacillus iners* (*L. iners*) in cervical cancer produces lactate, which rewires cancer cell metabolism to become resistant to chemoradiotherapy [20]. Gu et al. discovered that intratumoral bacteria promoted colorectal cancer liver metastasis by enhancing the production of lactate and its lactylation of target genes involved in immune suppression, inhibiting antitumor activities [21]. An oral squamous cell carcinoma (OSCC) tissue resident microbiota species *F. Nucleatum* was identified in the invasive margins of the lesions and was found to drive OSCC progression by enhancing glucose transporter 1 (GLUT1) accumulation on OSSC cell membranes and subsequent lactate production, which, in turn, induced macrophage M2-like polarization to become tumor-associated macrophages (TAMs) [22]. By analyzing lung adenocarcinoma (LUAD) microbiome data sets, Deng et al. developed lung-resident microbial score (LMS), glycolysis–lactate score, and glycolysis–lactate signature for predicting TME phenotypes, prognosis of LUAD patients, and response to immunotherapy [23]. Since different microbiota may have different glycolysis–lactate signatures, defining these signatures with the aid of advanced omics technologies can be clinically beneficial for predicting specific cancer type risks, prognosis, and response to therapeutics. Since the tumor microbiome is generally at low biomass and the intratumoral bacteria are mostly intracellular and are present in both cancer and immune cells [24], the crosstalk between the tumor microbiota and cancer cells, stromal cells, and immune cells for lactate production, as well as the overlapping functions of the lactate produced by microbiota and those produced by cancer cells, stromal cells, and immune cells will need to be further investigated.

### 1.5. Systemic Lactate Generation

A pioneering study using animal models by Warburg and colleagues confirmed that normal tissues under regular living conditions do not release lactate into circulation but clear lactate from the blood [4]. Normal cells only produce lactate when their oxygen (O_2_) supply or respiration is stopped, suggesting that the source of blood lactate could be exclusively from erythrocytes [4].

At human physiological levels, gut microbes can produce lactate, which is normally converted to propionate, butyrate, or acetate by certain microbes and does not accumulate in the gut lumen [25]. Carbohydrate overload, however, can lead to the excessive generation of lactate by gut microbes that enters the circulation. In addition, the gut microbiota homeostasis can be disrupted under disease conditions, resulting in lactate accumulation and transfer into the bloodstream. In a recent study, Leija et al. observed a postprandial two-wave blood lactate rises in healthy humans [26]. The first rise was due to the enteric postprandial lactate shuttle (PLS), which occurred prior to any glucose increase. The second rise in blood lactate was systemic PLS that coincided with a glucose increase driven by hepatic glucose release. These mechanisms of lactate production in aerobic tissues may, according to the authors, function as a means of carbohydrate carbon distribution and metabolism [26], an extension of the lactate shuttle (LS) theory, where lactate functions in delivering oxidative and gluconeogenic substrates as well as in cell signaling [27]. Interestingly, it was reported that a human fecal bacterial strain (Ec-TMU) produces indole-3-lactic acid (ILA), which downregulated glycolysis, NF-κB, and hypoxia-inducible factor (HIF) signaling activities involved in CCL2/7 production as well as inflammatory macrophage accumulation; it also inhibited colitis [28]. Such nonconventional means of lactate production and function challenge us to evaluate the role of lactate from a broader perspective.

Systemically, lactate functions at both physiological and pathological levels. For instance, skeletal muscle cells produce and release a substantial amount of lactate into circulation after intense exercise [29,30]. Psychosocial stress can also increase blood lactate levels [31]. The circulating lactate in the blood is an energy source for the brain, heart, and other tissues that require an immediate energy supply to cope with physical stress. The heart, for instance, utilizes lactate as an energy source and signaling molecule to maintain normal cardiovascular functions [32]. On the other hand, circulation is an efficient buffering mechanism to quickly remove lactate from the production site and distribute it for use or disposal by all available means in the body. Under certain noncancerous disease conditions, such as cardiovascular diseases, pulmonary hypertension or fibrosis, atherosclerosis, infection, trauma, kidney diseases, asthma, and liver disease, lactate is produced via glycolysis in the diseased tissues and released into circulation [33,34,35].

Systemic lactate metabolism is suggested to play three main roles, namely as a major energy source, as a gluconeogenic precursor, and as a signaling molecule that can induce autocrine-, paracrine-, and endocrine-like effects and, hence, is called “lactormone” [27,36]. Thus, lactate production and function at systemic levels are likely a defense response mechanism of the body for keeping up with energy needs and resolving stress conditions.

**Figure 3:**
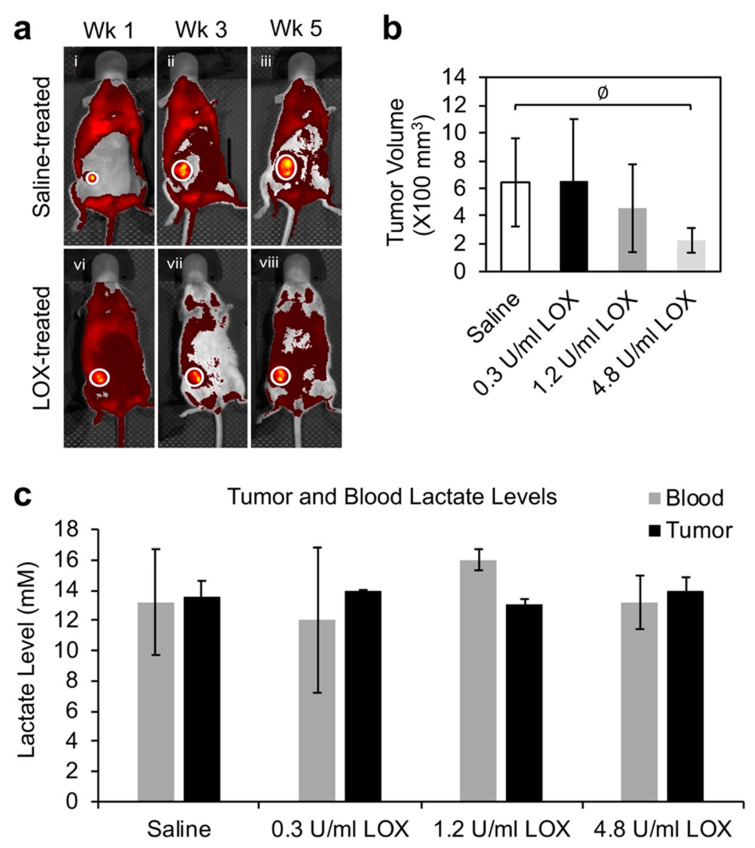
Systemic LOX administration impaired tumor growth in mice without affecting tumor lactate levels. (**a**) IVIS image of mice treated with saline or 4.8 U/mL LOX via tail vein injection. (**b**) Tumor size quantification. The excised tumors at Wk5 were measured with calipers, and their sizes in terms of volume were calculated with the equation (length × width^2^)/2. ∅: *p* = 0.055. (**c**) Tumor and circulating lactate levels in the mice treated with saline or LOX (previously unpublished data).

Since LOX is able to effectively deplete lactate in cell cultures and inhibit cancer cell growth [6,10], we recently examined the effect of systemic LOX injection (via tail vein) on mouse mammary tumor and circulating lactate levels. A substantial but nonsignificant tumor growth inhibition was observed at the endpoint of the experiment when high doses of LOX was used (Figure 3a,b). Interestingly, both systemic lactate (withdrawn from mouse heart) and tumoral lactate (collected by centrifugation of diced tumor tissues) remained at comparably high levels, similar to the saline-treated group, after LOX treatment (Figure 3c), suggesting that the systemic lactate pool is large and even high doses of LOX at sublethal levels are barely able to decrease systemic lactate levels. The high lactate levels observed in the saline-treated (baseline) group could be due to the active conditions of the animals, since they were acclimated before the experiment and the injections were conducted weekly. It could also be possible that the animal body could not dispose of the highly produced lactate during the experimental period.

## 2. TME Lactate Functions

### 2.1. Acidification of the TME

At the physiological pH of about 7.4 [37,38], lactic acid is predominantly converted to its conjugate base, lactate [1]. Lactate released from muscle activities can be converted to glycogen either in muscle tissues by a non-Cori cycle mechanism or in the liver or kidney via the Cori cycle [39]. Compared to a burst release of lactate during physical activities, tumors exhibit high rates of proliferation and glycolysis, which locally generate a significant amount of lactate that is an energy source for the growing tumors, leading to the establishment of a perpetual lactate-producing mechanism and an acidic extracellular environment that can range from pH 6.3 to 6.9 [40], a phenotype called acidification. With the great demand for energy by growing tumors and their active metabolism, it is unlikely that any cells within the TME would go down the glycogen synthesis route to provide glucose for further consumption. A recycling use of lactate, pyruvate, or other intermediate metabolic products could be more efficient to meet the needs of cancer cells and their supporting cells. TME acidification enables cancer progression by promoting cancer cell proliferation, migration, and invasion [41]. These cellular phenotypes are largely the result of genetic mutations that favor carcinogenesis, enhanced metabolism to match the carcinogenic progression, and the establishment of a TME that undermines immunosurveillance and promotes tumor development [13]. The acidic TME induces normal cell death and ECM degradation while presenting no harm to cancer cells, which have normal or alkaline intracellular pH (7.1–7.8) [42], making space for cancer cell proliferation and tumor formation [43].

Additionally, the low TME pH enhances the production of new actin filaments, increasing the binding of cancer cell surface integrins to ECM components and facilitating cell migration [44]. The increased acidity in the ECM increases the numbers and sizes of tumor cell “invadopodia”, which facilitates amoeboid movement and promotes tumor invasion [45]. Furthermore, increasing the alkalinity of the TME directly inhibits tumor invasion [43], indicating that the acidic nature of the TME is essential for cancer cell local invasion. TME acidification also facilitates the activation of enzymes, such as matrix metalloproteinase 9 (MMP-9), hyaluronidase-2, and cathepsin B, which degrade the ECM and promote invasion [40,46]. The degradation of the ECM by these enzymes may promote the activation of growth factors, such as vascular endothelial growth factor (Vegf), leading to cancer growth and survival [47]. The upregulation of Vegf through HIF-1α signaling also stimulates angiogenesis, the process of new blood vessel formation from pre-existing ones [48]. This process ensures a steady supply of oxygen and nutrients to growing tumors, while also creating abnormal and leaky vessels that promote metastasis.

An acidic TME also facilitates cancer cell evasion of immune suppression. This is achieved overall by inhibiting the proliferation of tumor-suppressing T cells and stimulating the proliferation of immunosuppressing regulatory T cells (Tregs), which promote cancer progression [49,50,51,52]. As a result of the acidic TME-inspired tumor advancement and deficient TME adaptive immunity, cancer cells within a tumor tend to develop resistance to anticancer drugs [53]. The TME acidification-associated immunopathy and drug resistance will be discussed in more detail in the following sections.

### 2.2. TME Lactate Signaling

**Figure 4:**
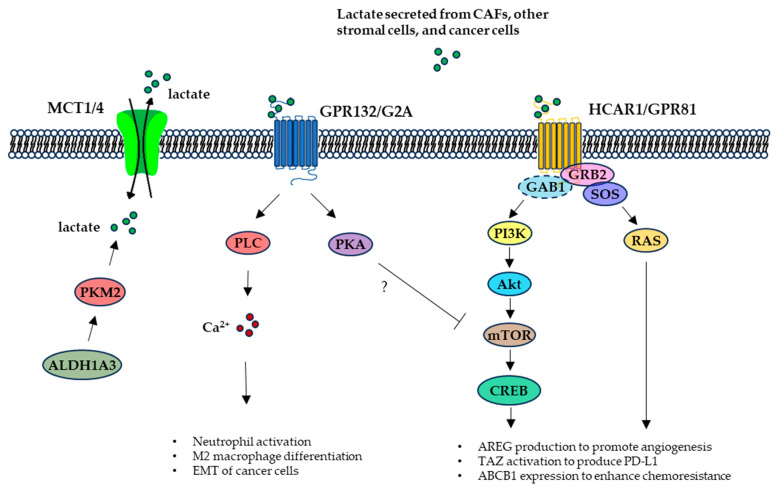
TME lactate signaling. Lactates produced from ALDH1A3/PKM2 activation or secreted from CAFs, other stromal cells, and cancer cells can either shuttle through MCT1/4 lactate transporters or functions as a signaling molecule by binding to GRP132 or HCAR1. Downstream effects of these signaling include activating immune cells and inducing their differentiation, stimulating cancer cell EMT, triggering angiogenesis, enhancing chemoresistance, and enabling cancer cell escape from immune surveillance. The question mark represents yet to be verified signaling pathway triggered by lactate.

Blood lactate levels can reach 15–25 mM in 3–8 min after intense to maximal exercise [54]. This exercise-induced blood lactate accumulation was suggested as a primary stimulator for growth hormone secretion [55] and an example for the myokine, exerkine, or lactormone signaling effects of lactate [36,56]. In addition to dynamic shuttling through cell membrane MCT1 and MCT4 transporters for metabolic demands, lactate may trigger cellular biological responses via two cell membrane G-protein-coupled receptors (GPCRs), namely the hydroxycarboxylic acid receptor 1 (HCAR1, also called GPR81) and the G protein-coupled receptor 132 (GPR132, also known as G2A) (Figure 4).

Lactate-directed activation of the lactate receptors related to oncogenesis or cancer progression is poorly understood. However, a couple of studies focusing on the role of HCAR1 in malignancy allude to a lactate–HCAR1 signaling switch, in an autocrine or paracrine way, that could potentiate cancer cell thriving in the TME and facilitate their ability to invade surrounding tissues and spread to distant sites [57,58,59]. For instance, HCAR1 was shown to mediate pro-angiogenic mediator amphiregulin (AREG) production to promote angiogenesis via the phosphatidylinositol 3-kinase (PI3K)/AKT serine/threonine kinase (Akt)-cAMP response element-binding protein (CREB) pathway in breast cancer cells (Figure 4), though the involvement of lactate in this regulation remained unclear in the study [57]. In a HCAR1-deficient breast tumor animal model, a paracrine regulation of cancer cell-secreted lactate on dendritic cell (DC) surface HCAR1 activation and subsequent suppression of DC presentation of tumor-specific antigen to other immune cells was observed [58]. Consistently, lactate activation of HCAR1 in lung cancer cells further activated the transcription coactivator TAZ, which interacted with the transcription factor transcriptional enhanced associate domain (TEAD) to induce programmed death-ligand 1 (PD-L1) expression that could inhibit cytotoxic T cell killing of the cancer cells [57,60,61]. Additionally, HCAR1 expression levels correlated with pancreatic tumor growth and metastasis in animals and was shown to be required for lactate stimulated MCT expression in pancreatic cancer cells [59]. Furthermore, HCAR1 was observed to be involved in lactate-stimulated expression of ATP-binding cassette sub-family B member 1 (ABCB1), a key mediator of chemoresistance, in cervical cancer cells [61]. Similarly, HCAR1-mediated recruitment of the C-C motif chemokine receptor 2 positive (CCR2+) immunosuppressive polymorphonuclear myeloid-derived suppressor cells (PMN-MDSCs) to the TME was observed in colorectal cancer cells, and reserpine inhibition of HCAR1 activation by lactate decreased CCR2+ PMN-MDSCs recruitment and enhanced antitumor immunity and cancer cell sensitivity to programmed cell death protein 1 (PD-1) antibody treatment [62]. Recently, we discovered a HCAR1 signaling cascade, where TME lactate secreted from breast cancer cells triggered cellular HCAR1 association with the PI3K and RAS signaling pathway components, such as growth factor receptor-bound protein 2 (GRB2), SOS Ras/Rac guanine nucleotide exchange factor 1 (SOS1), Kirsten rat sarcoma viral oncogene homologue (KRAS), GRB2-associated binding protein 1 (GAB1), and PI3K, for the activation of the oncogenic RAS and PI3K signaling pathways [10] (Figure 4). Depleting the TME lactate with LOX induced dissociation of the HCAR1 protein complexes and degradation of the HCAR1-associated proteins [10], highlighting the essential role of lactate–HCAR1 signaling in cancer cell survival and disease progression and the complexity of the signaling events downstream of lactate-triggered HCAR1 activation. Though HCAR1 could be a good target for cancer therapeutics, the wide expression of HCAR1 in various tissues, such as brain, adipose tissue, retina, and blood vessels and the broad distribution of lactate through circulation, may limit the therapeutic options targeting HCAR1.

The role of GPR132 as a selective lactate receptor remains controversial. This is because GPR132 was also suggested to be a receptor or effector for lysophospholipids (lyso-PLs) involved in Gα13- and Gαs-mediated HeLa cell apoptosis and in Galphai/phospholipase C (PLC) signaling for calcium flux in neutrophils [63,64] and may be a receptor for oxidized free fatty acids [65]. Nonetheless, GPR132 agonist activation of a GPR132-Gs-PKA pathway interfered with mTOR signaling and promoted acute myeloid leukemia (AML) cell differentiation, suggesting a therapeutic approach to AML treatment [66]. GPR132 was shown to be required for tumor-associated macrophage (TAM, M2 type) activation and sensing of TME lactate/acidic conditions to promote breast cancer cell proliferation, migration, invasion, and metastasis [67].

### 2.3. TME Lactate Regulation of Gene Transcription and Expression

As an essential energy source, a metabolic product, a major shuttle molecule voyaging across the MCT1 and the MCT4 lactate transporters, and a signaling-triggering ligand, lactate can undoubtedly induce changes in the transcription and expression of genes whose products function to coordinate the biological responses of the cells to the TME lactate level and overall signaling alterations. The lactate-stimulated broad gene expression changes, dubbed lactate-related gene signatures (LRGSs), have been identified in colorectal cancer, nasopharyngeal cancer, cutaneous melanoma, breast cancer, glioma, and gastric cancer using bioinformatics analysis of the gene expression datasets in the corresponding databases [68,69,70,71,72,73]. Overall, the major LRGS are related to cellular metabolism, oxidation process, cytokine signaling, immune response, and ECM organization. Monitoring the alterations of certain LRGS in a specific type of cancer prior to and after therapeutic treatment may have prognostic value [68,69,70].

It is interesting that lactate was shown to induce HCAR1 expression by upregulation of the transcription factor Snail, which complexed with enhancer of zeste homolog 2 (EZH2) and signal transducer and activator of transcription 3 (STAT3) and induced STAT3 binding to the HCAR1 promoter for its transcription [74]. This regulation suggests a feedback mechanism of the lactate-triggered cellular signaling to enhance the membrane HCAR1 capacities and activities and amplify HCAR1 downstream biological effects. Consistent with the tumor-promoting role of lactate, SW620 colon adenocarcinoma cells exposed to lactate displayed decreased sensitivity to the DNA-damaging chemotherapeutic drug cisplatin, coinciding with an increased expression of genes involved in DNA repair, oxidative stress response, cell adhesion and proliferation, and decreased expression of tumor suppressor genes [75].

CAFs are major tumor stromal cells, fundamental building blocks of the TME, and the chief craftsman for the establishment of an optimal tumor ECM, which provides structural support and biochemical ligands for cancer cell proliferation, migration, invasion, and metastasis [76,77,78]. It was shown that TME lactate downregulates CAF expression of p62 (also known as sequestosome 1 or SQSTM1), a suppressor of CAF activation and tumor progression, by decreasing the NAD+/NADH ratio and subsequent inhibition of poly(ADP-ribose)-polymerase 1 (PARP-1) activity that is required for activation of c-FOS, c-JUN and AP-1, the transcriptional complex for p62 gene expression [79]. On the other hand, CAF-secreted lactate promoted the prostate cancer cell expression of collagen genes, whose protein products were hydroxylated by lactate-mediated mechanisms and activated discoidin domain receptor 1 (DDR1) to promote the invasive traits of the cancer cells [80]. As stromal cells living in the TME, TAMs are undoubtedly under the influence of TME lactate. Shi et al. reported that lactate induced immunosuppressive gene expression by promoting histone H3K27 acetylation, resulting in the repression of macrophage pro-inflammatory function while having no effect on the canonical LPS/TLR4/NF-κB and p38 MAPK signaling-mediated pro-inflammation response [81]. In contrast, in a chronic social defeat stress (CSDS) mouse model, supplying lactate to the animal restored hippocampus histone deacetylases HDAC2 and HDAC3 protein levels and their deacetylase activities, thereby promoting the resilience of the mice to CSDS and implicating an antidepressant function of lactate [82]. These lines of evidence suggest that lactate may switch on different modes of histone modifications, such as acetylation or deacetylation, required for specific sets of gene expression in response to specific stimuli in different tissues.

Furthermore, lactate produced by stromal cells, such as CAFs [83], or aldehyde dehydrogenase 1 family member A3 (ALDH1A3)-induced PKM2 tetramerization [84] activities in addition to sources described above preferentially maintains cancer cell stemness in many cancer types, including triple-negative breast cancer [85], liver cancer [86,87], glioma [88], glioblastoma [84], and colorectal cancer [89]. Increased levels of lactate maintain cancer stem cells (CSCs) by suppressing differentiation, inducing dedifferentiation, and increasing proliferation through various mechanisms, which include activating the Hippo pathway via the discs large homolog 5 (DLG5)/cullin 3 (CUL3)/ mammalian Ste20-like kinase 1 (MST1)/ Yes-associated protein 1 (YAP1) axis [83], increasing histone acetylation and activating MYC in a bromodomain-containing protein 4 (BRD4)-dependent manner [90], in addition to overexpressing LDHD, which catabolizes D-lactate for energy demand required for self-renewal through the cyclin-dependent kinase 7 (CDK7)-YAP-LDHD axis [91].

Histone lactylation (Kla) describes a posttranslational modification (PTM) mechanism wherein lactate induces epigenetic modification, i.e., the addition of a lactyl group to the ε-amino group of a lysine residue of histone, which directly modulates gene transcription [92]. Importantly, Kla is directly regulated by aerobic glycolysis-generated lactate and was suggested to initiate the expression of homeostatic genes, such as arginase 1 (Arg1) and vascular endothelial growth factor A (Vegfa), associated with M2-like macrophage characteristics and wound healing in the late phase of M1 macrophage polarization [92]. Kla has been observed in different types of cancer cells [33] and implicated in the progression of various cancers. For example, elevated Kla of H3K18 was found in the promoter region of the YTH N6-methyladenosine RNA-binding protein 2 (YTHDF2), which binds to the m6A sites of the tumor suppressor PER1 and TP53 mRNAs for their degradation, and YTHDF2’s enhanced transcriptional expression was associated with the progression and poor prognosis of ocular melanoma [93]. In non-small cell lung cancer (NSCLC) cells, lactate treatment triggered increased Kla in the promoters of the glycolytic enzyme hexokinase 1 (HK-1) and the TCA cycle enzyme isocitrate dehydrogenase (NAD(+)) 3 non-catalytic subunit gamma (IDH3G), resulting in decreased or increased expression of the metabolic response genes associated with the Kla of HK-1 and IDH3G, respectively [94]. It has also been shown in CSCs that there is a global elevation of lactylation in H3 histone at H3K9, H3K24, and H3K56 sites that is crucial for maintaining their stemness [86,87,95].

Interestingly, a non-histone lactylation was observed in NSCLC using lactylomic and metabolomic profiling [96]. The authors found that cellular lactate increased apolipoprotein C2 (APOC2) lactylation at K70 (lactyl-APOC2-K70), which induced free fatty acid (FFA) release into the TME, extracellular lipolysis, and Treg cell accumulation associated with immunotherapy resistance and tumor metastasis [96]. Mechanistically, non-histone lactylation was recently shown to be mediated by alanyl-tRNA synthetase 1 (AARS1), an intracellular lactate sensor and lactyltransferase mediating global lysine lactylation [97]. AARS1 binds to lactate to produce lactate–AMP in an ATP-dependent manner and covalently conjugates lactate to TP53 K120 and K139 residues to impair its binding to p53-responsive genes and, thus, the tumor-suppressing function of p53 [97]. The authors further showed that β-alanine could disrupt lactate binding to AARS1, impede p53 lactylation, and inhibit tumorigenesis in animal models [97]. Additionally, lactate can be converted to lactyl-CoA for histone and non-histone lactylation [33]. A non-enzymatic acyl transfer of the D-lactate moiety from lactoylglutathione (LGSH) to protein Lys residues to generate a “LactoylLys” modification on glycolytic enzymes was observed [98]. This PTM mechanism feeds back to glycolysis regulation under hyperglycemic or Warburg-like conditions.

There are many lactylated proteins which play major roles in CSCs. Aldolase A (ALDOA) expression is upregulated in many cancer cells, and ALDOA increases intracellular lactate levels and induces H3K14 lactylation [95]. ALDOA typically binds to dead box deconjugate enzyme 17 (DDX17) in the cytoplasm [86]. ALDOA lactylation at the K230 and K322 sites causes DDX17 release from ALDOA, allowing DDX17 entry into the nucleus and binding to SRY-box transcription factor 2 (SOX2) to activate their target genes to maintain cancer cell stemness. Another lactylated protein that is crucial in CSCs is polypyrimidine tract-binding protein 1 (PTBP1) [88]. PTBP1 K436-lactylation inhibits PTBP1 binding to a tripartite motif containing-21 (TRIM21), an E3 ubiquitin ligase, and prevents its proteasomal degradation. Furthermore, K436-lactylated PTBP1 binds and stabilizes 6-phosphofructo-2-kinase/fructose-2,6-biphosphatase 4 (PFKFB4) mRNA, increasing glycolysis.

Enhanced chemoresistance is found to be associated with the lactylation of several key proteins. H4K12 lactylation by p300 upregulates the catalytic subunit of glutamate-cysteine ligase (GCLC) expression and inhibits ferroptosis [89]. X-ray repair cross-complementing protein 1 (XRCC1), which is involved in base excision and single-strand break repair, is lactylated at the K247 site, promoting its nuclear translocation and enhancing DNA repair [84]. Nijmegen breakage syndrome protein 1 (NBS1) is lactylated at the K388 site, and this lactylation is crucial for the MRE11-RAD50-NBS1 (MRN) complex to form [99]. This allows the MRN complex to accumulate at DNA double-strand breaks to begin homologous recombination in order to carry out repairs.

**Figure 5:**
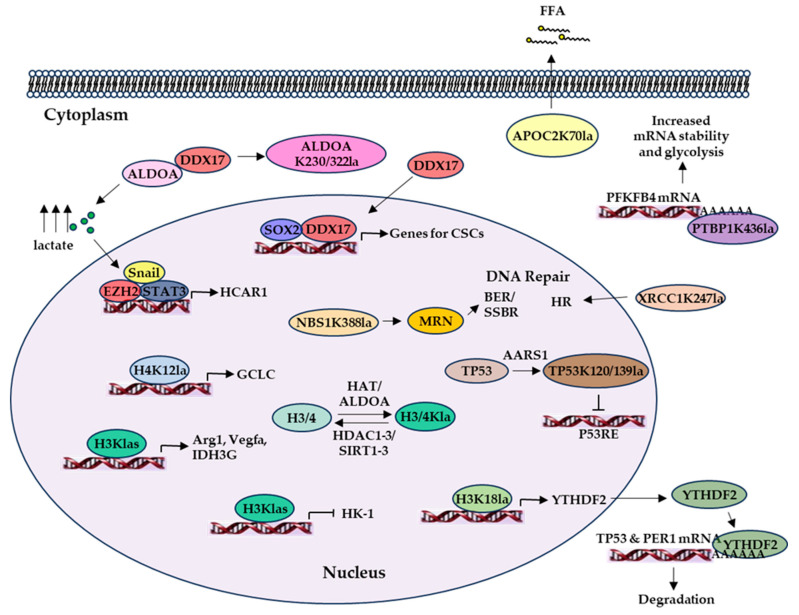
Lactate and lactylation regulate gene expression and protein and fatty acid functions. Increased intracellular lactate levels can activate Snail/EZH2/STAT3 to initiate HCAR1 transcription. Histone lactylation activates gene transcription, such as for Arg1, Vegfa, IDH3G, YTHDF2, and GCLC, or inhibits HK-1 transcription. TP53K120/139 lactylation by AARS1 can inhibit the p53 response element, while increased production of YTHDF2 from H3K18la can cause degradation of TP53 and PER1 mRNA. PTBP1K436la can stabilize PFKFB4 mRNA to increase glycolysis. ALDOAK230/322la allows the release of DDX17 and its translocation into the nucleus to interact with SOX2 to activate genes important for maintaining cancer cell stemness. Lactylation of APOC2 at K70 increases secretion of FFA.

Collectively, lactylation demonstrates an overall tumor-promoting functionality, as summarized in Figure 5. It is worth noting that lactylation, like other PTMs, such as phosphorylation and acetylation, can be regulated by signaling and PTM enzymes, and even reversed by delactylation. For instance, hypoxic or bacterial exposure signals could induce lactylation [92], and the histone acetyltransferase p300 can promote lactate-induced histone lactylation [100,101]. Recently, HDAC1–3 and SIRT1–3 have been found to be delactylases, and HDAC3 can effectively delactylate both L- and D-lactyl lysine [102]. Thus, lactylation and delactylation of cellular biomolecules represent a counterbalance mechanism regulating the pro- and anti-tumor activities of cancer cells, stromal cells, and immune cells in the TME. Therapeutic approaches targeting lactylation or delactylation and their regulation may open new opportunities for effective cancer treatment [103].

### 2.4. Lactate Modification of the TME

**Figure 6:**
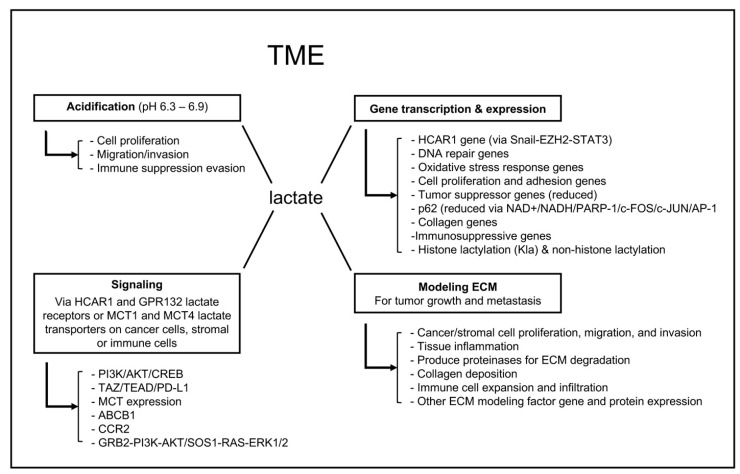
The major functions of TME lactate. The TME lactate is a pH tuner, signaling molecule, epigenetic and transcription regulator, and ECM modifier.

As a key TME and cellular metabolite, TME pH tuner, signaling ligand and transducer, epigenetic and gene transcription regulator, lactate directly or indirectly modifies the TME. First of all, lactate promotes cancer cell, stromal cell, and immune cell proliferation, migration, and/or invasion, as well as tissue inflammation and tumor angiogenesis [27,103,104]. These mass-increasing activities in the tissues directly and physically change the TME. On the other hand, lactate or the acidic TME that it produces can activate protein-degrading enzymes, such as metalloproteinases, cathepsin, and hyaluronidase, to destroy the ECM around a growing tumor [105]. In parallel, TME lactate accumulation enhances cancer or stromal cell collagen gene expression, collagen production and hydroxylation, and deposition to further alter the ECM structures toward a more tumor favorable TME [80]. The indirect modification of the TME by lactate involves its downstream effectors, such as its cell membrane transporters and receptors. It was reported that primary myelofibrosis (PMF) patient CD34+ cells produced excess lactate and overexpressed MCT1 and MCT4 lactate transporters, which promoted immunosuppressive Treg cell and myeloid-derived suppressor cell (MDSC) expansion and mesenchymal stromal cell (MSC) remodeling of the ECM [106]. Upregulation of the lactate receptor HCAR1 has been associated with cancer progression. HCAR1 was suggested to regulate the expression of genes, such as protocadherin 7 (PCDH7), EPH receptor A7 (EPHA7), and the delta-like canonical Notch ligand 4 (DLL4), involved in ECM remodeling, cell adhesion, and cell–cell interaction [107]. The above TME lactate functions collectively to have an effect on the overall TME setup to promote tumor progression (Figure 6).

## 3. Lactate and Local Immunity

TME lactate, low pH, and hypoxia are major factors allowing tumors to evade immune surveillance [108,109,110,111,112,113]. Lactate can promote either a pro-inflammatory or anti-inflammatory environment depending on the context of the disease [108,113,114]. Using T cells as an example, during a typical inflammatory response, extracellular lactate is transported inside CD4+ helper cells by SLC15A12 and cytotoxic CD8+ cells by SLC16A1 (aka MCT1) [115] and/or generated intracellularly by LDHA [116]. Intracellular lactate inhibits T cell migration, causing T cells to linger at the site of inflammation [115]. In addition, it promotes pro-inflammatory responses by inducing IFN-γ expression via increased histone acetylation [116] and posttranslational 3′UTR-mediated regulation [117]. Furthermore, lactate promotes Th17 differentiation by inducing pyruvate kinase M2 isoform (PKM2) to dimerize and translocate into the nucleus to phosphorylate STAT3 [114]. Thus, STAT3 in combination with retinoic acid receptor-related orphan receptor-γt (RORγt) activates IL-17 transcription.

T effector cells are highly sensitive to glucose levels, since T cell activation by CD28 stimulation increases glucose uptake through glucose transporter GLUT1 and glycolysis [118]. High levels of lactate can decrease glucose uptake and increase intracellular NADH levels leading to inhibitory feedback on glycolysis [12,114]. Therefore, the low glucose levels and high lactate concentrations typically found in the TME cause T cell inactivation and decrease IFN-γ production by inhibiting the nuclear factor of activated T cells (NFAT), thus decreasing tumor surveillance [112,113]. Furthermore, MCT11 on CD8+ T cells enhances lactate uptake and inhibits their cytotoxic regulation of tumor growth [119]. This inhibition, which leads to CD8+ T cell exhaustion, is mediated by C-X9-C Motif Containing 1 (CMC1), a mitochondrial electron transport chain complex IV chaperon protein [120]. Lactate increases CMC1 levels by inducing USP7, which deubiquitinates CMC1 and, thus, promotes its stability. Tumors can also secrete endothelial cell-specific molecule 1 (ESM1) in a lactate-dependent manner [121]. ESM1 can then enhance stearoyl-CoA desaturase 1 (SCD1) in CD8+ T cell to suppress its antitumor activity [121] by increasing oleic acid and esterified cholesterol, which is generated by acetyl-CoA acetyltransferase 1 (ACAT1) [122]. On the other hand, exogenously administered lactate in a pH neutral environment can instead augment anti-tumor function of CD8+ T cell by inhibiting activity of HDAC, resulting in enhanced acetylation at H3K27 of the T cell factor 7 (Tcf7) super enhancer locus and, thus, increased Tcf7 expression [123].

Treg cells utilize OXPHOS instead of glycolysis for their energy consumption [124]. In fact, high glucose levels and increased glucose uptake impair Treg cell function, and Treg cells instead upregulate metabolic pathways that utilize lactate via MCT1 and HCAR1 [125]. The link between lactate and OXPHOS in Treg cells is mediated by α-1,3-Mannosyl-Glycoprotein 2-β-N-Acetylglucosaminyltransferase (MGAT1) [126]. Intracellular lactate activates X-box binding protein 1 (XBP1), leading to increased MGAT1 transcription and translocation into mitochondria and, thus, initiate N-glycosylation of progranulin (GRN) and hypoxia-upregulated 1 (HYOU1) to promote OXPHOS. Moreover, forkhead box P3 (FOXP3), a major factor for natural Treg cells, enhances Treg cell survival and its suppressive functions in a low glucose, high lactate environment [127]. This enhanced Treg cell function is partially mediated by lactate-dependent ubiquitin-specific peptidase 39 (USP39), a component of the RNA splicing machinery, to augment cytotoxic T-lymphocyte antigen 4 (CTLA-4) expression in a FOXP3-dependent manner and, thus, to prevent cytotoxic T cell function [128]. Furthermore, lactate enhances lactylation of Lys72 in membrane-organizing extension spike protein (MOESIN), which promotes Treg cell generation by improving the interaction of MOESIN and TGF-β receptor I and, thus, enhancing mothers against decapentaplegic homolog 3 (SMAD3) signaling [129]. Tumors can also secrete CX3CL1 chemokine by lactate-induced signaling of HCAR1 to promote the direct recruitment of Treg cells into the TME [130].

As for TAMs, it was initially discovered that MDSCs residing in the TME are pleiotropic and consist of monocytes and macrophages mixed with both M1 pro-inflammatory and M2 immunosuppressive characteristics [131]. Later, it was determined that there were distinct populations of myeloid cells with monocyte-derived M1 macrophages and monocytic MDSCs that differentiate into M2 macrophages within the TME [132]. In M1 macrophages, increased intracellular lactate activates PKM2, which leads to pSTAT3 and the stabilization of HIF-1α, promoting IL-6 and IL-1β [133,134]. Interestingly, IL-1β secretion by TAM in a lactate-dependent manner can upregulate PD-L1 expression in tumor cells to enhance their escape from immune surveillance [135]. PD-L1 can be expressed on macrophages, DCs, T cells, and tumor cells, and PD-L1 expression is increased by the direct binding of PKM2 and HIF-1α to HRE sites on the PD-L1 promoter [136]. Therefore, lactate and IL-1β can increase PD-L1 levels in a positive feedback loop mediated by the activation of PKM2.

**Figure 7:**
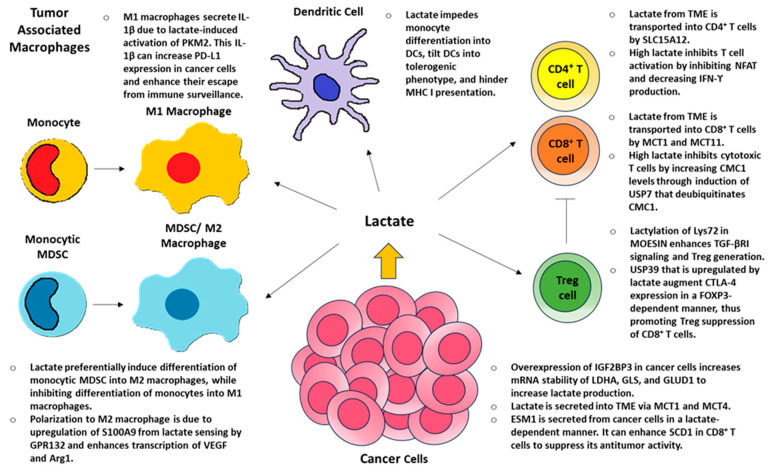
Lactate in the TME promotes overall immunosuppression. Cancer cells overexpress IGF2BP3 to increase lactate production, and lactate is secreted into the TME via MCT1 and MCT4. Lactate is then transported into various immune cells within the TME through various transporters, such as MCT1, MCT11, and SLC15A12, and also interacts with HCAR1 and GPR132. The overall effect is to prevent a pro-inflammatory environment by inhibiting IFN-γ-producing CD4+ T cells and preventing monocytes from differentiating into M1 macrophages and DCs, while promoting anti-inflammatory environment by inducing monocytic MDSCs into M2 macrophages, promoting Treg differentiation and enhancing their function, and inhibiting cytotoxic CD8+ T cell function through the various mechanisms described above. This allows cancer cells to escape from immune surveillance.

Lactate also activates IL-23 transcription in TLR-stimulated monocytes/macrophages, promoting a Th17 profile [137]. On the other hand, lactate also inhibits glycolysis in monocytes, suppressing differentiation into a M1 TNF-α-secreting phenotype, and lactate also helps differentiate monocytic MDSCs into M2 macrophages [49,138,139,140,141]. M2 macrophages secrete IL-10, IL-13, and TGFβ and, thus, are involved in immune regulation and tumor survival [40]. This shift in M2 macrophage polarization due to the upregulation of S100 calcium-binding protein A9 (S100A9) [142] is facilitated by lactate sensing by GPR132 [67] and the expression of Vegf and Arg1 through HIF-1α [139,143] or, alternatively, by activation of ERK-STAT3 signaling pathway [144]. In addition, lactate impedes monocyte differentiation into DCs [145,146] and, furthermore, tilts DCs towards a tolerogenic phenotype as well as hindering MHC class I antigen presentation, preventing anti-tumor response [147,148]. The effects of lactate on the immune response within TME are summarized in Figure 7.

## 4. TME Lactate and Influences on Therapy

### 4.1. TME Lactate Effect on Therapeutic Resistance

Mediating lactate levels to facilitate cancer treatment has been clinically investigated at multiple levels. The primary areas targeted include inhibiting lactate formation via LDHA (gossypol/AT-101), a reduction in oxidative phosphorylation (metformin), decreasing lactate formation via the inhibition of pyruvate dehydrogenase kinase (dichloroacetate), and disrupting lactate transport (AZD3965) [149,150,151,152,153,154,155,156,157,158,159,160,161].

Gossypol is a naturally occurring compound derived from cotton plants. Its more active R-(-)-enantiomer, R-(-)-gossypol acetic acid, or AT-101, has been utilized for clinical trials. Renner et al. has nicely summarized the outcomes of several of these trials [156]. Single-agent phase I and phase II trials of AT-101 as a single-agent therapy were mostly disappointing. The authors reported on seven phase I and phase II trials encompassing advanced adrenal cortical carcinoma, recurrent small cell lung carcinoma, castration-resistant prostate cancer, refractory metastatic breast cancer, recurrent glial tumors, metastatic adrenal cancer, and advanced cancers not otherwise specified [156]. The dose administered ranged from 10 mg BID to 70 mg daily. The one trial in advanced cancers not otherwise specified included biphasic weekly dosing that ranged from 30 mg to 180 mg [156]. Grade 3 or 4 toxicities included troponin elevation, hypokalemia, AST/ALT elevation, fatigue, nausea, vomiting, anorexia, and small bowel obstruction. Of these seven single-agent trials, five were terminated due to futility or poor response rates [149,156,159,161]. In contrast, in the trial of metastatic adrenal cancers, 3 out of 18 patients demonstrated a partial response, despite having failed other chemotherapy regimens [156].

Renner et al. also reviewed nine phase I/II clinical trials and a single phase III trial of AT-101 used with the addition of adjuvant chemotherapy and/or radiotherapy [156]. The sole phase III study was a double-blind randomized study of AT-101 (20 mg daily) or placebo with docetaxel and cisplatin for the treatment of NSCLC. Thirty-one patients were enrolled in each arm. There was no significant difference in serious adverse events between groups. Although there was no significant difference in progression-free survival (PFS) or overall survival (OS), there was a trend in favor of the AT-101 group (PFS 7.43 vs. 4.9 months; OS 18.37 vs. 14.7 months) [156]. The largest multi-agent study performed was a phase II double-blind trial of AT-101 (40 mg BID) or placebo with docetaxel in metastatic castration-resistant prostate cancer [156]. The AT-101 group experienced a higher rate of grade III adverse events, including pulmonary embolus, cardiac events, lymphopenia, neutropenia, and neuropathy. This led to a higher rate of patients discontinuing therapy or undergoing dose reductions in the AT-101 arm. No significant differences were identified in OS, PFS, or reduction in PSA levels. The authors indicated that a potential OS benefit was noted in a particularly high-risk subgroup, with an OS of 19 months vs. 14 months [156]. Of the 10 trials reviewed, only 2 demonstrated a modest clinical benefit.

Metformin is a commonly used drug in the treatment of type 2 diabetes mellitus. Metformin alters cellular metabolism by accumulating within mitochondria and directly inhibiting oxidative phosphorylation complex I, a step that decreases ATP formation and shifts cells toward energy conservation. It can actually increase lactate production and induce cancer cell apoptosis [150]. Curry reported on 39 patients with HNSCC treated between diagnostic biopsy and surgical resection with metformin [150]. Thereafter, tissues were assessed for metabolic markers and apoptosis. Of the 39 patients, 31 showed increased caveolin 1 (CAV1) staining after metformin treatment. Low stromal CAV1 is commonly found in HNSCC and can upregulate MCT4 in cancer associated fibroblasts [150]. Interestingly, MCT4 was not upregulated in these patients. Increased carcinoma apoptosis was noted following metformin treatment, and lactate production increased 2.4-fold [150]. Since the treatment course of metformin was quite short, at an average of 13 days, no information on response rate, survival, or recurrence was available [150].

Dichloroacetate (DCA) decreases blood and intracellular lactate concentrations via the inhibition of pyruvate dehydrogenase kinase. In so doing, DCA preferentially promotes oxidative metabolism rather than the production of lactate [151,152,155,158]. A phase I trial of DCA in the treatment of recurrent malignant brain tumors showed no dose-limiting toxicities [151]. Two of eight patients experienced grade 0/1 paresthesia, but DCA was well-tolerated overall. In a single-agent phase II clinical trial, Tian et al. evaluated the relationship between DCA concentration, glutathione S-transferase zeta 1 (GSTZ1, the primary DCA metabolizer) genotype, side effects, and patient response in six patients with multiple myeloma [158]. DCA was given orally for 3 months. Only one of six patients showed a partial response to DCA. No dose-limiting toxicities were encountered. Another single-agent phase II trial of DCA was conducted in previously treated stage IIIB/IV NSCLC or stage IV breast cancer [152]. Six NSCLC patients enrolled, but two withdrew within 1 week; only one breast cancer patient enrolled. Further enrollment was halted after these seven patients, as the trial was terminated for safety concerns: within 1 week of initiating DCA treatment, a patient died of an unknown cause, while another died of a pulmonary embolus. The authors concluded that NSCLC patients did not benefit from DCA. No conclusion could be drawn from the sole breast cancer patient, although stable disease was maintained for 8 weeks, followed by disease progression [152]. The authors also evaluated the role of DCA in 54 NSCLC cell lines in normoxic and hypoxic conditions with and without the addition of cisplatin and docetaxel chemotherapy. In cell lines, DCA as a single agent was not effective. Under normoxic conditions, DCA did not enhance the tumor inhibition of either chemotherapy agent. Under hypoxic conditions, however, DCA and cisplatin showed synergistic induction of tumor cell death [152]. These pre-clinical data led the authors to conclude that future evaluation of DCA should be considered when given with other chemotherapy agents [152]. Multiple treatment modalities were evaluated in a phase II trial of locally advanced HNSCC [155]. In this trial, Powell et al. randomized patients to receive DCA or placebo in conjunction with cisplatin and radiation therapy. There were no significant differences in grade 3/4 adverse events, but the DCA arm was notable for having a significantly higher complete response rate (71.4% vs. 37.5%) [155].

AZD3965 is an inhibitor specific to MCT1. To date, AZD3965 has only been evaluated in a single phase I clinical trial. Halford et al. performed a dose escalation study in patients with advanced cancer; forty patients were enrolled [153]. Dosing ranged from 5 mg to 30 mg once daily or 10 or 15 mg BID. The most common mild grade 1/2 adverse events included retinopathy, fatigue, anorexia, and constipation. Grade 3 adverse events included troponin elevation in one patient, acidosis in one patient, and ocular changes in five patients. Overall, these clinical studies targeting tumor lactate production, transport, and metabolism have shown the limited efficacy of the drugs that were tested.

### 4.2. Pre-Clinical Studies and Novel Directions

Pre-clinical studies have evaluated various mechanisms of lactate manipulation [162], some of which are quite novel, incorporating the use of bicarbonate transporters [163], lactate transporter blockade [164,165,166,167,168], lactate depletion [169], and lactate modulating nanomedicines [169]. Cappellesso investigated the inhibition of the most abundant bicarbonate transporter, SLC4A4, as a means of mitigating TME acidification and decreasing lactate production via a reduction in glycolysis in pancreatic ductal adenocarcinoma (PDAC) cells [163]. The authors developed mouse Panc02 and KPC pancreatic cells engineered with a doxycycline inducible CRISPR–Cas9 system and either a single guide RNA targeting SLC4A4 or a non-targeting guide RNA (control). While this genetic targeting did not influence cell growth or apoptosis in vitro for either cell line, SLC4A4 targeting showed reduced tumor growth in subcutaneous and orthotopically injected Panc02 and KPC tumors. In vitro studies demonstrated a reduction in bicarbonate uptake and lactate production in both cell types, as well as a decrease in the intracellular pH and an increase in the extracellular pH. Of note, SLC4A4-targeted Panc02 tumors, but not KPC tumors, showed an increase in CD8+ T cells. The CD8+ T cells in KPC tumors, while not increasing in number, were more activated owing to the increased expression of CD69 and IFN-γ. The authors concluded that the decreased growth of SLC4A4-targeted pancreatic tumors was due to increased immune activation and reduced immunosuppression. This was further confirmed when SLC4A4 targeting was combined with immune checkpoint blockade (anti-CTLA4 and anti-PD-1). The control mice had a median survival of 32 days, while the SLC4A4-targeted mice treated with immune checkpoint blockade were all alive and healthy at day 80 [163].

While the use of MCT1 inhibition was discussed previously in the context of clinical trials of agent AZD3965, MCT4 inhibition has also been evaluated in pre-clinical studies. Choi et al. developed MCT4 antisense oligonucleotides (ASOs) and tested them in a tissue microarray model of castration-resistant prostate cancer [165]. Increased MCT4 expression was associated with higher Gleason score prostate cancer and an earlier time to relapse. Furthermore, elevated MCT4 expression was seen in tumors from men treated with neoadjuvant hormone therapy for >6 months, indicating a role of increased MCT4 expression in the development of castration resistance. ASOs were designed and tested in PC-3 cell lines to confirm the inhibition of proliferation. The most promising candidates were found to inhibit MCT4 in PC-3 cell lines in a dose-dependent manner. A similar effect was noted when the ASOs were tested on additional cell lines C4-2, DU145, and LNCaP. MCT4 ASOs were found to downregulate genes involved in glycolysis (GAPDH, PGK1, PGAM1, ENO1, and LDHA). The growth of xenografts of PC-3 cells in nude mice were inhibited by MCT4 ASOs. Finally, these nude mice treated with MCT4 ASOs were found to have increased levels of NK cells and an increase in CD3 staining, an indicator of NK cell activation [165]. Further translation to the clinical environment seems promising.

Babl evaluated the prospect of immune checkpoint blockade with single-agent MCT4 inhibition and combined MCT4 and MCT1 inhibition in colorectal carcinoma (CRC) [164]. In CRC, MCT4 expression, not MCT1, was most important for CRC patient survival according to transcriptome data from 326 colon cancer and 186 rectal cancer samples obtained from The Cancer Genome Atlas database. In a 3D tumor spheroid model of HCT116 CRC cells, MCT4 blockade decreased lactate secretion, but not tumor growth, even with the addition of MCT1 blockade. With the addition of peripheral blood leukocytes and immune checkpoint blockade to this 3D HC116 CRC cell tumor spheroid model, MCT4 blockade alone or with MCT1 blockade did not improve upon the effect of the immune checkpoint blockade alone. In an in vivo MC38 mouse model, however, single MCT4 and immune checkpoint blockade prolonged survival, delayed tumor growth, and increased tumor cell pH, tumoral leukocyte infiltration, and T cell activation [164].

The prospect of dual inhibition of MCT1 and MCT4 was also evaluated in feline and human oral squamous cell carcinoma by Khammanivong et al. [168]. A combined MCT1 and MCT4 inhibitor, MD-1, was used to treat human HNSCC cell lines as well as feline oral squamous cell carcinoma (FOSCC) cell lines and was found to decrease cell viability in a dose-dependent manner. Increased intracellular lactate levels were noted in the HNSCC cell line, but not the FOSCC cell lines after MD-1 treatment. In vivo, MD-1 prolonged OS in an orthotopic model of FOSCC [168]. Draoui et al. investigated a novel family of lactate flux inhibitors, namely 7-aminocarboxycoumarins (7ACC1 and 7ACC2), in a variety of cancer cells expressing MCT1, MCT4, or both [166]. When compared with the combined MCT1/MCT2 inhibitor AR-C155858, 7ACC1 and 7ACC2 were able to block lactate influx in SiHa cervical cancer cells, HeLa cervical cancer cells, and FaDu HNSCC cells. In tumor cells expressing MCT1 and MCT4 lactate transporters, the 7ACC compounds were not able to prevent lactate efflux but were able to inhibit lactate influx. The 7ACC compounds showed activity against tumor growth in cervical SiHa tumors, CRC HCT116 tumors, and orthotopic breast MCF-7 tumors [166].

Although the MCT1 inhibitor AZD3965 has been previously discussed in the context of clinical trials, Huang et al. described a novel modification of this drug to make a new nanodrug compound, AZD-UPS NP [167]. AZD-UPS NP is composed of AZD3965 loaded inside ultra-pH-sensitive nanoparticles. At normal physiological pH, the compound remains intact but disassembles to release the active drug component (AZD3965) when exposed to an acidic pH. In TC-1 cancer cell-bearing mice, AZD-UPS NP or standard AZD3965 was combined with an immune checkpoint blockade (anti-PD-1). The nanodrug led to significant growth inhibition and improved survival compared to the control group and the AZD3965 group. This was duplicated in the melanoma cell line B16F10 [167]. The engineering of lactate-modulating nanomedicines represents a novel and promising strategy, with several new compounds being developed [162,169].

## 5. Conclusions

Production-wise and function-wise, TME lactate is more than just a glycolytic metabolite. TME lactate is weaponized as a multifunctional molecule that can provide energy to fuel tumor progression, acidify the TME to favor the voracious propagation of malignant cells, serve as ligands for its receptors and transporters and signaling relay runners to amplify its impacts, act as an epigenetic and transcriptional modifier to alter gene expression in favor of tumor growth, suppress tissue immunity to aid cancer cell escape immune checkpoints, and equip cancer cells with resistance to radio-, chemo-, or immunotherapy. Therefore, the battle to eliminate the lactate effect in the TME and defeat tumor progression is deemed to be challenging and yet rewarding if an efficient way is discovered.

Both increasing and decreasing TME lactate have been studied as cancer therapeutic strategies, as demonstrated in the LDHA or MCT inhibitor and Metformin clinical trials. The collective outcomes of these trials are underwhelming. While many of the therapeutic strategies targeting lactate production, transport, or metabolism work perfectly in vitro or in animal models, they have failed to work in the human body, which is more complex and has a large systemic or TME pool of lactate. This is similar to an effective LOX inhibition of cancer cell or tumor growth in cultures and animal models [6,10,170,171,172], respectively, where local LOX administration seems to work well. However, systemic LOX injection failed to decrease both whole body and tumor lactate levels and insignificantly inhibited tumor growth in animals (Figure 3). This phenomenon raises the questions of how effective TME local lactate depletion can be and how long the depletion can last because the systemic pool of lactate may fill in the TME lactate depletion deficit quickly. It also challenges certain observed cancer cell- or tumor-inhibition effects considered to be the results of lactate production or metabolic inhibition. In other words, are those phenotypic inhibitions direct outcomes of the targeted mediators’ lactate-modulating functions or other indirect roles associated with cancer cell survival and tumor growth?

Another unavoidable question is how the TME lactate pool and systemic lactate pool are coordinated during carcinogenesis and progression. A more clinically important aspect related to this question is how much lactate is a product of physical stress, cancer-associated complications, and therapeutic side effects that also drains into the pool of the TME lactate, drives disease progression, and can be eliminated to facilitate the cure of cancer. Based on clinical observations, it seems the therapeutic approaches targeting the TME lactate and its activities are naturally an integral and beneficial part of a combined clinical therapy that can maximize cancer treatment efficacies.

## Data Availability

Sharing of the published data is subject to federal and institutional policies and to request should be made to the corresponding authors.

## Abbreviations

The following abbreviations are used in this manuscript:

AARS1	Alanyl-tRNA synthetase 1
ALDH1A3	Aldehyde dehydrogenase 1 family member A3
ABCB1	ATP-binding cassette sub-family B member 1
ACAT1	Acetyl-CoA acetyltransferase 1
ALDOA	Aldolase A
AML	Acute myeloid leukemia
APOC2	Apolipoprotein C2
AREG	Amphiregulin
Arg1	Arginase 1
ASOs	Antisense oligonucleotides
BRD4	Bromodomain-containing protein 4
CAFs	Cancer-associated fibroblasts
CAV1	Caveolin 1
CCR2	C-C motif chemokine receptor 2
CDK7	Cyclin-dependent kinase 7
CMC1	C-X9-C Motif Containing 1
CRC	Colorectal carcinoma
CREB	cAMP response element-binding protein
CSCs	Cancer stem cells
CSDS	Chronic social defeat stress
CTLA-4	Cytotoxic T-lymphocyte antigen 4
DC	Dendritic cell
DCA	Dichloroacetate
DDR1	Discoidin domain receptor 1
DDX17	Dead box deconjugate enzyme 17
DLG5	Discs large homolog 5
DLL4	Delta-like canonical Notch ligand 4
ECM	Extracellular matrix
EPHA7	EPH receptor A7
ESM1	Endothelial cell-specific molecule 1
EZH2	Enhancer of zeste homolog 2
FFA	Free fatty acid
FOSCC	Feline oral squamous cell carcinoma
FOXP3	Forkhead box P3
GAB1	GRB2-associated binding protein 1
GCLC	Glutamate–cysteine ligase
GLS	Glutaminase
GLUD1	Glutamate dehydrogenase 1
GLUT1	Glucose transporter 1
GPCRs	G-protein-coupled receptors
GPR132	G protein-coupled receptor 132 (aka G2A)
GRB2	Growth factor receptor-bound protein 2
GRN	Progranulin
HCAR1	Hydroxycarboxylic acid receptor 1 (aka GPR81)
HGF	Hepatocyte growth factor
HIF1-α	Hypoxia-inducible factor 1 subunit alpha
HK-1	Hexokinase 1
HNSCC	Head and neck squamous cell carcinoma
HYOU1	Hypoxia-upregulated 1
IDH3G	Isocitrate dehydrogenase (NAD(+)) 3 non-catalytic subunit gamma
IGF2BP3	Insulin-like growth factor 2 mRNA-binding protein 3
ILA	Indole-3-lactic acid
Kla	Histone lactylation
KRAS	Kirsten rat sarcoma viral oncogene homologue
*L. iners*	*Lactobacillus iners*
LDHA	Lactate dehydrogenase A
LGSH	Lactoylglutathione
LMS	Lung-resident microbial score
LOX	Lactate oxidase
LRGS	Lactate-related gene signature
LS	Lactate shuttle
LUAD	Lung adenocarcinoma
lyso-PLs	Lysophospholipids
MCT	Monocarboxylate transporters
MDSC	Myeloid-derived suppressor cell
MGAT1	α-1,3-Mannosyl-Glycoprotein 2-β-N-Acetylglucosaminyltransferase
MMP-9	Matrix metalloproteinase 9
MOESIN	Membrane-organizing extension spike protein
MRN	MRE11-RAD50-NBS1
MSC	Mesenchymal stromal cell
MST1	Mammalian Ste20-like kinase 1
NBS1	Nijmegen breakage syndrome protein 1
NFAT	Nuclear factor of activated T cells
NSCLC	Non-small cell lung cancer
OSCC	Oral squamous cell carcinoma
OXPHOS	Oxidative phosphorylation
p62	Sequestosome 1 (aka SQSTM1)
PARP-1	Poly(ADP-ribose)-polymerase 1
PCa	Prostate cancer
PCDH7	Protocadherin 7
PD-1	Programmed cell death protein 1
PDAC	Pancreatic ductal adenocarcinoma
PD-L1	Programmed death-ligand 1
PFKFB4	6-phosphofructo-2-kinase/fructose-2,6-biphosphatase 4
PI3K	Phosphatidylinositol 3-kinase
PKM2	Pyruvate kinase M2 isoform
PLC	Phospholipase C
PLS	Postprandial lactate shuttle
PMF	Primary myelofibrosis
PMN-MDSCs	Polymorphonuclear myeloid-derived suppressor cells
PTBP1	Polypyrimidine tract-binding protein 1
PTM	Posttranslational modification
RORγt	Retinoic acid receptor-related orphan receptor-γt
ROS	Reactive oxygen species
S100A9	S100 calcium-binding protein A9
SCD1	Stearoyl-CoA desaturase 1
SLC1A5	Sodium-dependent solute carrier family 1 member 5
SMAD3	Mothers against decapentaplegic homolog 3
SOS1	SOS Ras/Rac guanine nucleotide exchange factor 1
SOX2	SRY-box transcription factor 2
STK11/LKB1	Serine/threonine kinase 11/liver kinase B1
TAMs	Tumor-associated macrophages
Tcf7	T cell factor 7
TEAD	Transcriptional enhanced associate domain
TKIs	Tyrosine kinase inhibitors
TME	Tumor microenvironment
Tregs	Regulatory T cells
TRIM21	Tripartite motif containing-21
USP39	Ubiquitin-specific peptidase 39
Vegf	Vascular endothelial growth factor
XBP1	X-box binding protein 1
XRCC1	X-ray repair cross-complementing protein 1
YAP1	Yes-associated protein 1
YTHDF2	YTH N6-methyladenosine RNA-binding protein 2
